# Influence of femoral broach shape on stem alignment using anterior approach for total hip arthroplasty: A radiologic comparative study of 3 different stems

**DOI:** 10.1371/journal.pone.0204591

**Published:** 2018-10-05

**Authors:** Cécile Batailler, Camdon Fary, Elvire Servien, Sébastien Lustig

**Affiliations:** 1 Department of Orthopaedic Surgery, Lyon North University Hospital, Lyon, France; 2 Department of Orthopaedic Surgery, Western Health, Melbourne, Australia; 3 Australian Institute for Musculoskeletal Science (AIMSS), The University of Melbourne and Western Health, St. Albans, VIC, Australia; University of Umeå, SWEDEN

## Abstract

**Background:**

Malalignment of the femoral stem in total hip arthroplasty (THA) can detrimentally affect outcome. Poor preparation of the femur intraoperatively is an important cause of stem malalignment.

**Purposes:**

The objective was to compare coronal alignment of three different stems using three different broaches.

**Methods:**

Retrospective study of three groups of 60 patients following primary THA via direct anterior approach, by the same surgeon, between January 2015 and January 2016. Each group had a similar designed stem (Corail Depuy, Targos Lepine or Meije Tornier). Groups were matched for age, body mass index, gender, side, neck shaft angle and indications. The significant difference between groups was the broach shape. Broaches for the Corail and Meije stems had a prominent shoulder laterally, while the broach of the Targos stem had a rounded less prominent shape laterally. Coronal alignment was determined radiologically at 2 months.

**Results:**

The mean varus was significantly lower for the Targos stems (1.1° +/-0.8) compared to the Corail (2.3° +/-1.5) and Meije stems (1.9° +/-1.2) (p<0.0001). There were significantly less Targos stems with varus greater than 3° (1.7%, n = 1) compared to the Corail (40%, n = 24) and Meije stems (20%, n = 12) (p<0.001).

**Conclusion:**

A femoral broach with a prominent lateral shoulder when performing a THA via direct anterior approach will increase the risk of varus femoral stem alignment compared to a less laterally prominent broach.

## Introduction

Femoral alignment is considered important for both survivorship and outcome in total hip arthroplasty (THA). Multiple studies have demonstrated varus stems are associated with poor clinical outcomes and increased complications e.g. aseptic loosening, secondary subsidence [1, 2] and thigh pain [3, 4]. Earlier studies were predominantly cemented stems [5–9] or the first uncemented stems [1, 10, 11]. Of these complications only loosening has been reported recently as not a risk factor [12, 13]. Regardless, if preoperative planning alignment of the stem is not varus but the post-operative is then malposition or malalignment has occurred.

Several factors can influence stem positioning, in particular the anatomical femoral bone shape [14], minimally invasive surgery [15], surgical approach [16], implant shape and the instruments for femoral preparation [17]. Optimal femoral preparation with appropriate instruments is crucial for appropriate alignment of the stem. Several unique instruments have been developed to optimize femoral preparation, particularly aggressive rasps for the lateral edge of the proximal femoral shaft [17]. Using the direct anterior approach (DAA), shape of the femoral broach appears to be an important factor in stem positioning, in particular for difficult cases. THA using the DAA has increased rapidly internationally over “traditional approaches” (i.e. anterolateral or posterior). In regions or hospitals of Europe it is the standard approach. Despite this very few studies have reported the impact of different broaches on femoral alignment with DAA.

The surgeons involved in this study use DAA for all primary THA. The exception is when significant femoral shortening or correction is required as part of the femoral preparation (e.g. femoral shortening osteotomy of greater than 3cm for a high dislocated congenital dysplastic hip when the preoperative plan is to return hip anatomical center of rotation). The posterior approach is used in this situation.

The objective of this study was to compare the preoperative plan to the definitive coronal alignment of three different stems implanted relative to different broach geometry.

## Method

### Patients

This is a retrospective study comparing three groups of patients following primary THA via DAA. The inclusion criteria for the three groups were first 60 patients operated for primary THA via DAA by the same surgeon between January 2015 and January 2016 using a Corail stem (Depuy), Targos stem (Lepine) or a Meije stem (Tornier). The surgeon was experienced in THA via DAA and is his routine approach for primary THA.

Exclusion criteria were previous surgery or trauma on the operated hip, femoral congenital deformities and femoral neck fractures.

The demographic characteristics are summarized in the Table 1. The three groups were matched for age, body mass index (BMI), gender, operated side, neck shaft angle and etiologies of osteoarthritis. Indications, femoral morphologies and surgeon experience were the same for all groups. The DAA is the standard approach in this department and the surgeon was not in the learning curve for this technique.

Intraoperative complications were recorded. At two-month routine follow up standard AP and lateral X-rays was taken and reviewed.

**Table 1:** Demographic characteristics of each group of patients according to the implanted stem (Corail, Meije, Targos).

	**Corail** Mean +/-SD Extremes		**Meije** Mean +/-SD Extremes		**Targos** Mean +/-SD Extremes		**P value**
**Patients**	60		60		60		
**Age (yo)**	71 +/-7 [56–85]		72 +/-8 [51–86]		71 +/-8 [56–89]		NS
**BMI (Kg/m^2^)**	27 +/-5 [17–38]		26 +/-4 [19–34]		27 +/-5 [17–38]		NS
**Gender (M)**	26 (43%)		16 (27%)		22 (37%)		NS
**Side (R)**	27 (45%)		36 (60%)		29 (48%)		NS
**Neck shaft angle (°)**	128 +/-5.6 [116–143]		129 +/-5.6 [116–140]		128 +/-4.7 [118–142]		NS
**Etiology**							
**Essential**	53 (88%)		51 (85%)		54 (90%)		NS
**Post-traumatic**	0		1 (1.7%)		1 (1.7%)		
**Dysplasia**	2 (3.3%)		6 (10%)		3 (5%)		
**Osteonecrosis**	4 (6.7%)		1 (1.7%)		2 (3.3%)		
**Inflammatory**	1 (1.7%)		1 (1.7%)		0		

(SD: Standard Deviation; yo: years old; NS: No Significant; M: Male; R: Right)

### Ethical approval

All procedures performed in studies involving human participants were in accordance with the ethical standards of the institutional and/or national research committee and with the 1964 Helsinki declaration and its later amendments or comparable ethical standards.

The Advisory Committee on Research Information Processing in the Field of Health (CCTIRS) approved this study in Paris under number 2018-AO1653-5. For this type of study formal consent is not required (retrospective and anonymous study). All data were fully anonymized before their access. The ethics committee did not require an informed consent.

### Implants

**Fig 1:**
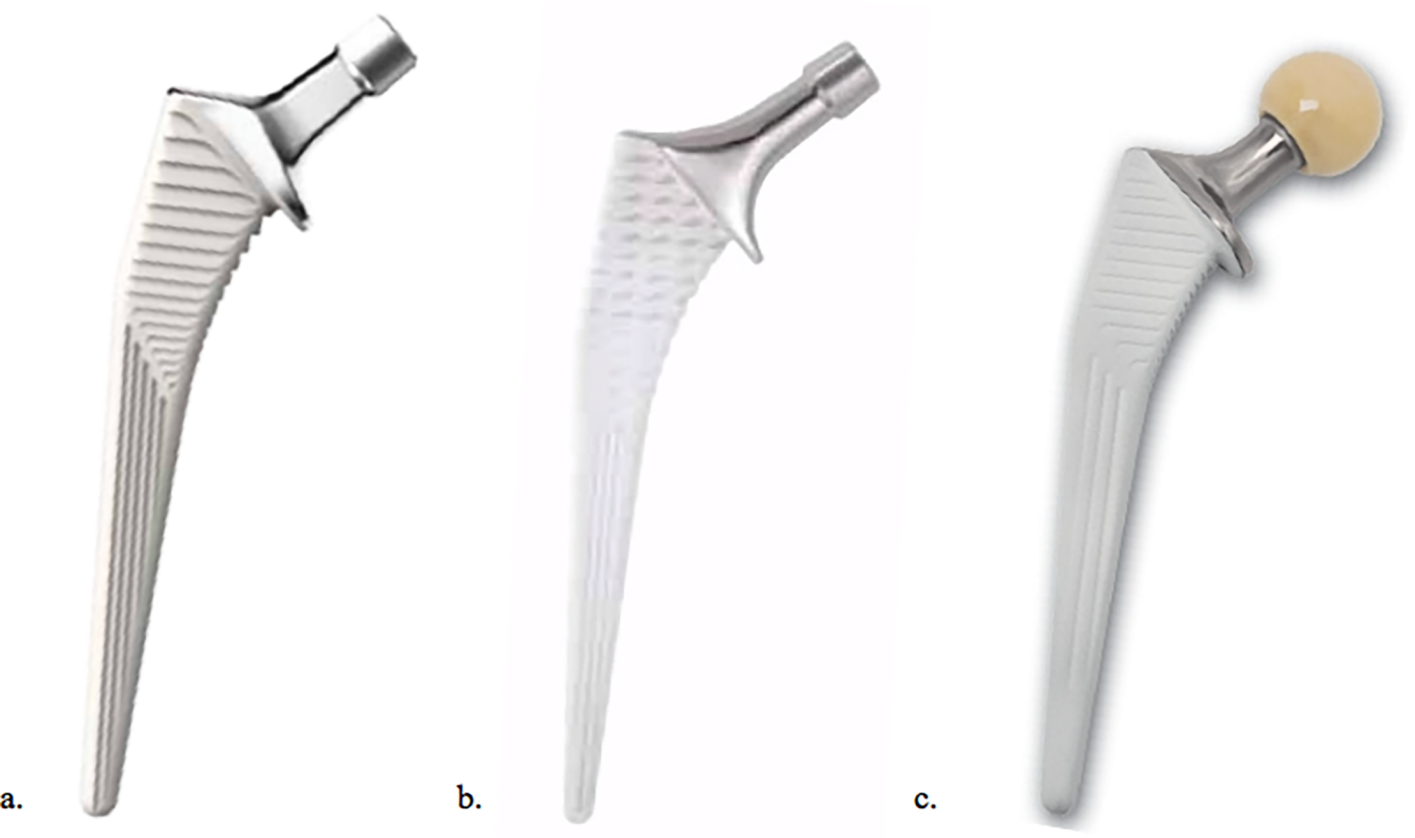
The three stems used during this study. a. Corail Depuy; b. Meije Tornier; c. Targos Lepine. The design includes a fully hydroxyapatite coating non-porous forged titanium alloy stem, with a proximal trapezoidal/quadrangular cross-section and a tapered distal stem. All stems had a collar.

The three cementless stems (Corail Depuy, Meije Tornier, Targos Lepine) are of similar design. Each had a nonporous fully hydroxyapatite coating on a forged titanium alloy stem (Fig 1). The design similarities included a proximal trapezoidal/quadrangular cross section intended to resist axial/torsional stresses and promote initial stability, tapered distal stem which is quadrangular and provide a decreasing stiffness gradient. All stems in this study had a collar.

**Fig 2:**
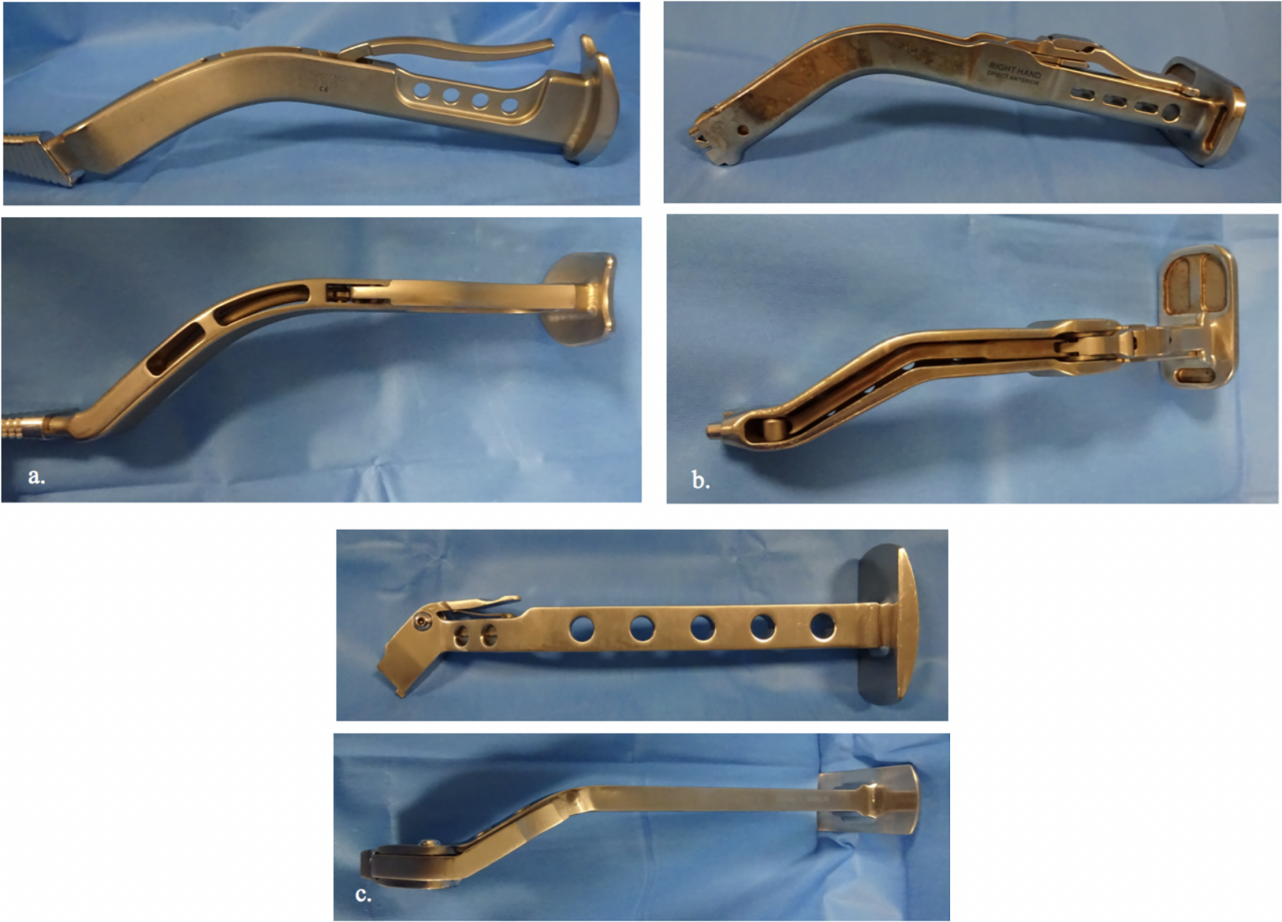
The three broach handles used in this study with lateral and superior views. (a. Corail Depuy; b. Meije Tornier; c. Targos Lepine). All had a double offset with the same curve, allowing a femoral preparation in a satisfying axis, even with difficult exposures.

A double offset broach handle was used in all cases (Fig 2) to avoid contact with superficial soft tissue and retractors. The three broach handles had similar sagittal and coronal curves. The sagittal curve was 142° Corail stem, 134° Meije stem and 132° for the Targos stem. The coronal curve was 148° Corail stem, 152° Meije stem and 150° for the Targos stem.

**Fig 3:**
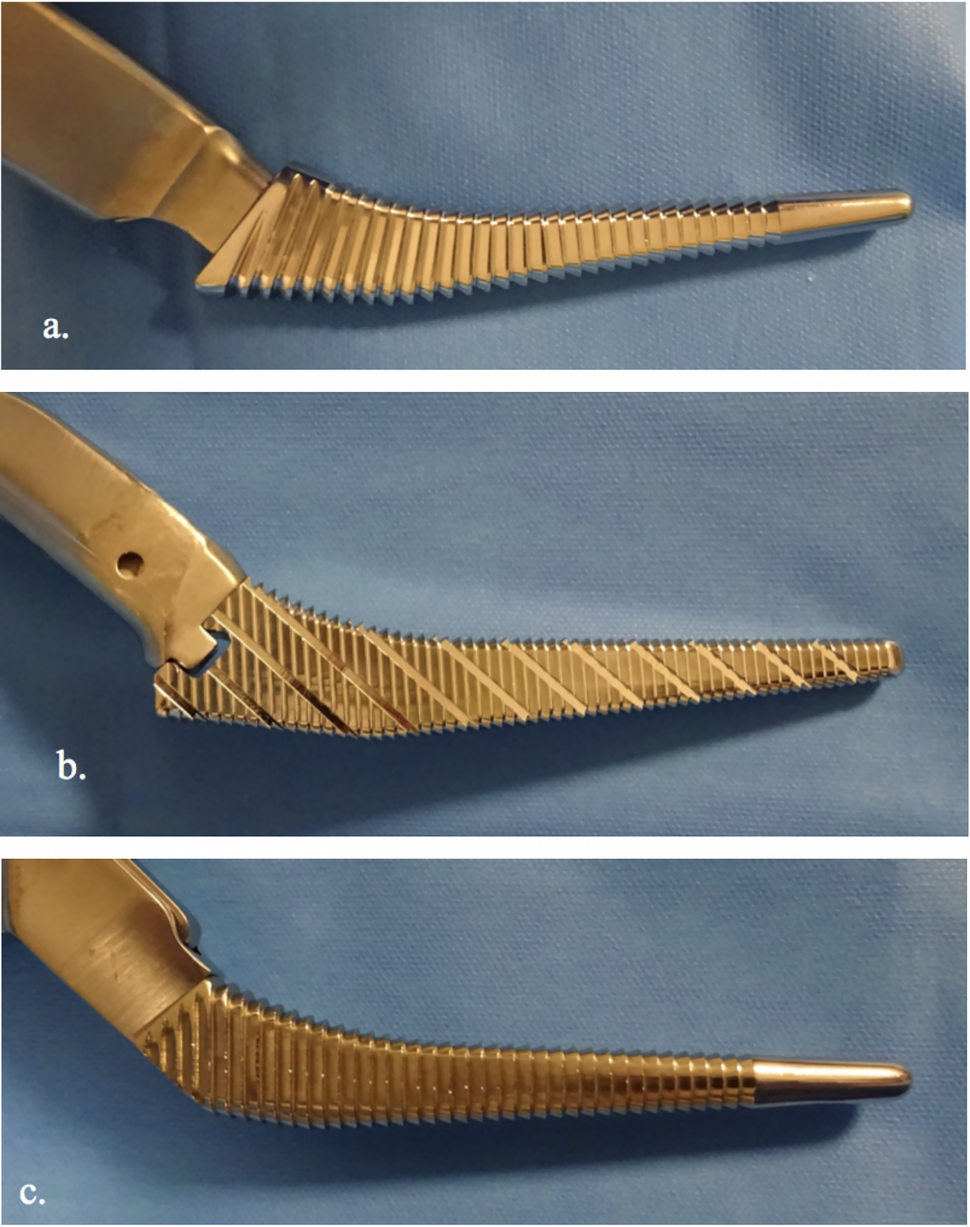
The three broaches used for the preparation femoral in this study. (a. Corail Depuy; b. Meije Tornier; c. Targos Lepine). The broach for the Targos stem presents a rounded back, in the continuity of the handle. The broaches for the Corail and Meije stems have a shoulder more or less marked, just before the handle.

The Corail and Meije broaches had a prominent shoulder laterally while the Targos stem broach laterally had a rounded shape (Fig 3). All broaches had identical attachment to the broach handle.

The aim was to prepare the femur appropriately to place all stems according to preoperative planning aligned with the coronal femoral axis.

### Measurements

All measurements were recorded by the same observer on an anteroposterior pelvic view radiograph at 2 months. Inter-observer reliability was assessed for 10 patients in each group. Radiological complications were recorded. Coronal alignment of the stem was determined by measuring the angle formed between the long axis of the prosthesis and the long axis of the femur. Positioning was recorded as positive for varus and negative for valgus. Coronal alignment greater (or equal) than 3° was considered a varus placed stem.

The width of stems in the proximal and distal part of the diaphyseal segment of each stem (ratio = width of distal part/ width of proximal part) were measured and compared. The Targos stem was designed with a larger distal portion as a method to regulate stem insertion distally and prevent malalignment.

### Statistical analyses

The continuous variables were averaged and reported with standard deviation and extremes. They were compared using Student t-test or Wilcoxon nonparametric test. The categorical variables were compared using a Fisher exact test. A p value <0.05 was considered statistically significant in each analysis. The intra-observer and inter-observer reliability of these measurements were evaluated by an intraclass correlation coefficient. The statistical analyses were performed with XLstat (2015 Addinsoft).

## Results

There were significantly less Targos stems placed in a varus position (1.7%, n = 1) compared to both the Meije (20%, n = 12) (p<0.001) and Corail stems (40%, n = 24) (p<0.001). The number of Meije stems in a varus position was significantly less than the Corail stems (p<0.05). All results are detailed in Table 2.

**Table 2:** Results of the frontal alignment of the stems after THA with three implants types. Comparison of the mean varus and of the proportion of stems with a varus superior to 3°, between the three stems (Corail, Meije, Targos).

	**Mean Varus** **Mean (°) +/-SD** **Extremes**	**p Value** **with Meije**	**p Value** **with Targos**
**Corail stem**	2.3 +/-1.5 [-1.4; 5.2]	NS	p <0.0001
**Meije stem**	1.9 +/-1.2 [-1; 4.5]	-	-
**Targos stem**	1.1 +/-0.8 [-1.1; 3.1]	p <0.0001	-
	**Varus ≥ 3°** **Nb patients (%)**	**p Value** **with Meije**	**p Value** **with Targos**
**Corail stem**	24 (40%)	p <0.05	p <0.0001
**Meije stem**	12 (20%)	-	-
**Targos stem**	1 (1.7%)	p <0.001	-

(SD: Standard Deviation; NS: No Significant)

**Fig 4:**
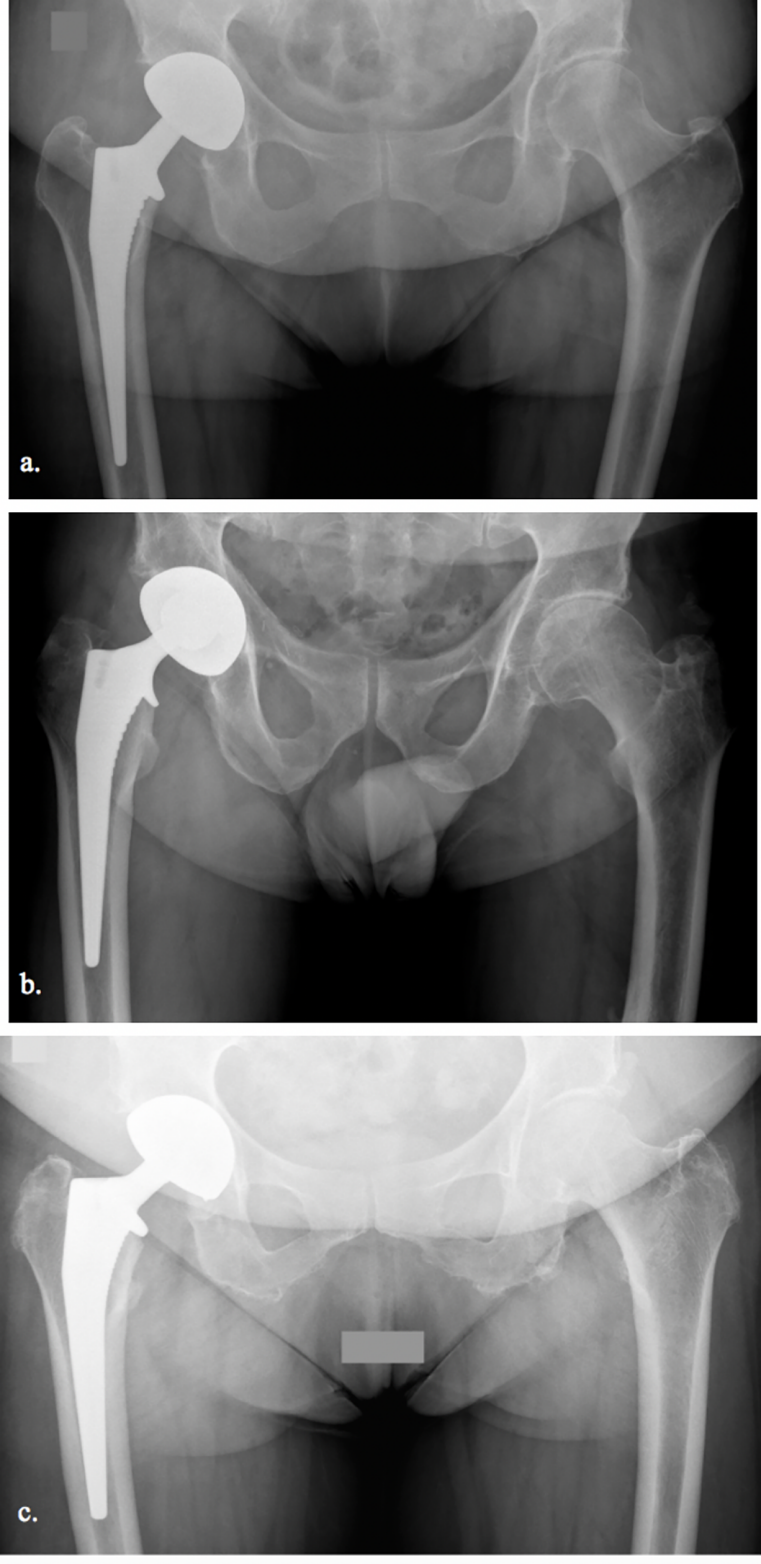
Postoperative pelvic AP views of three stems used in this study. a. Right THA with a Corail stem (varus of 4.3°). b. Right THA with a Meije stem (varus of 4°). c. Right THA with a Targos stem (only Targos stem with a varus positioning of 3.1°).

At 2 months no patient had a major complication requiring hospital admission or surgical revision. No patient had radiological subsidence of the stem. The mean varus of stems was 2.3° +/-1.5 Corail, 1.9° +/-1.2, Meije and 1.1° +/-0.8 Targos groups (Fig 4). The mean varus was significantly lower for the Targos stems compared to the Corail stems and the Meije stems (p<0.0001). The difference between the mean varus of the Corail and Meije stems was not significant (p = 0.07).

No patient had a valgus position greater than -3°. Only five patients had a valgus coronal alignment between -0.4° and -1.4° without significant difference between the three groups (1 Corail stem, 2 Meije stems, 2 Targos stems).

The mean ratio between the proximal part and the distal part of the diaphyseal segment of stems was different between the three stems, though not significantly (Corail stem: 0.271; Meije stem: 0.304; Targos stem: 0.335).

Radiological measurement of the stem alignment was reliable, with both a strong intraclass correlation (ICC = 0.86) and interclass correlation (ICC = 0.81).

## Discussion

The most important finding of this study is that there was a significantly higher rate of stems of a similar design placed in varus greater (or equal) than 3° when the femoral broach had a prominent lateral shoulder via DAA. This is the first study to compare varus positioning of different femoral stems relative to broach shape for DAA. This is important factor to be aware of when preparing the femur using a broach with a prominent lateral shoulder and may help in preventing varus alignment relative to preoperative planned alignment from occurring.

Murphy et al. studied the femoral alignment of 200 uncemented THA with the Corail hip system. They reported that varus positioning could sometimes be related to femoral morphology, particularly coxa vara deformity, and was not necessarily a technical failure [14]. However, in our study, the neck shaft angle was comparable between the three groups and did not explain our difference in the varus positioning. Insufficient preparation of the lateral edge of the femoral shaft or persistence of the lateral superior cortical bone of the femoral neck can cause varus displacement, particularly with a coxa vara deformity. With the DAA, the femoral preparation is sometimes more difficult and the risk of malalignment is slightly higher compared to posterior approach [16]. For these more challenging cases instruments for femoral preparation should be optimal.

The group with a less prominent lateral shoulder of the broach (Targos) resulted in only one stem in varus greater than 3° and a low mean varus relative to the preoperative plan and femoral axis. This less prominent broach allows work on the femoral shaft smoothly in a medial to lateral movement. This is because during early femoral preparation there is decreased initial contact with either/or the proximal lateral femoral shaft, lateral edge of greater trochanter and superolateral femoral neck cortical bone. Any or all of these three bone contact points can displace preparation and stem placement into varus. This is particularly important to be aware of in difficult cases when the initial broach may be placed in varus and not corrected with each successive increase in size until the definitive size is reached. The two other groups had a significantly greater number of stems in varus greater than 3°, with Corail greatest at 40%.

Further comparison between the broaches of the Targos (one stem (1.7%) in varus) to the Corail (24 stems (40%) in varus) was an increased aggressiveness or sharpness of the Targos. This would result in removal of bone with the Targos broach rather than impaction which occurred with the Corail during femoral preparation. A broach that impacts bone is forced away from harder cortical bone towards softer metaphyseal bone. If with initial broaching the hardest contact is prominent lateral cortical bone of either the greater trochanter or superior femoral neck, as described above, the broach could be forced into varus. If the first broach is in varus and not recognized then the malalignment will be propagated as broach size is increased sequentially.

The Targos stem compared to the literature had a very low varus rate (1.7%) where varus rates have been reported up to 25% [3, 18, 19]. While the Corail stem at 40% had a higher rate of stems in varus position than the literature, however the mean varus remained less than 3° for all three groups. Mean varus is rarely described in the literature, but has been reported up to 10° [13, 18].

Several complications have been described with a stem in varus, particularly thigh pain, femoral loosening, stem undersizing with postoperative subsidence [1–4, 10, 11, 20]. The effect of femoral positioning on long term survivorship and outcome is debated in the literature [12, 13, 18, 19]. Either way it should be considered optimal that the surgeon should be able to position the femoral implant according to their preoperative planning. In difficult cases (previous hip surgery or trauma, difficult exposure, severe osteoporosis….) the risk of having a different alignment to planning is increased. The design of surgical instruments contributes to this. Several instruments have been developed to optimize this positioning, particularly the aggressive rasps for the lateral edge of the proximal femoral shaft. But very few studies assessed the role of these instruments for alignment. Hjorth et al. have described the broaching preparation technique [17] and found increased migration when preparing the bone with compaction compared with broaching in cementless femoral stems.

This study had several limitations. It was retrospective and patients were not randomized in each group. Each group were matched with the choice of the implant by availability independent of femoral anatomy. Three similar but not identical designs of implants with their own specific instruments were assessed rather than only one design. The offset of the broach handles is slightly different between the three implants, but this difference is too small to explain the varus rate. The stem design is also a little different between the three (Targos stem has a larger distal portion than other two stems). However, there was no significant valgus positioning for the three stems, and the difference of the ratio of the proximal and distal diaphyseal part of each was not significant. Thus, we believe that the larger diaphyseal stem of the Targos is not the sole explanation for the different varus rate. It is a strength that this single surgeon study removes surgeon experience. The DAA could be a risk factor of varus positioning with inappropriate technique. The surgeon was experienced for this approach and the double offset broach handle is standard use in our department [21]. Our results should not be extrapolated to other surgical approaches. DAA is the standard approach in our department. Lastly, this study didn’t evaluate the clinical outcomes, because our objective was not to determine the best femoral stem or the impact of a femoral stem with varus positioning.

## Conclusion

Femoral alignment is an important parameter during THA implantation. Multiple factors can adversely affect the alignment, in particular the design of the instruments to prepare the femur. Comparing three stems and their instrumentation, this study showed that there were less stems in varus when a more aggressive broach that had a less prominent lateral shoulder was used via DAA. Femoral stem positioning is influenced by the design of the instruments used to prepare the femur. To be aware of this risk and anticipate will decrease the chance of a femoral stem in varus alignment using the DAA.

## Supporting information

S1 Table Study data. All anonymized data of this study are reported on a table in supporting information.
(XLSX)
